# Supplementary material for: Deletion of cftr Leads to an Excessive Neutrophilic Response and Defective Tissue Repair in a Zebrafish Model of Sterile Inflammation
Source: Front Immunol. 2020 Jul 31;11:1733. doi: 10.3389/fimmu.2020.01733 (PMC7412881; doi:10.3389/fimmu.2020.01733)
Supplement: Supplementary file 1 [file Data_Sheet_1.PDF]

# *Supplementary Material*

## Supplementary Figures

**Supplementary Figure 1. Generation of CFTR-depleted zebrafish**

(A) Generation of *cftr* null mutant in zebrafish using CRISPR-Cas9 gene editing. Schematic of the domain structure of CFTR and with a star indicating the CRISPR-Cas9 target site. Double guide RNA (gRNA) for CRISPR-Cas9-mediated stable *cftr* inactivation have been designed to effectively induce a deletion in *cftr* gene. The expected targeted deletion (127 bp) in F0 founder fish and germline transmission were detected and confirmed by PCR Analysis. Heterozygous carriers for the mutation are crossed to obtain *cftr* $^{-/-}$ homozygous mutant (#sh540). *cftr* -/- mutants are screening for impaired Kupffer's vesicle inflation at 8-somite stage. (B) Loss of CFTR causes Kupffer's vesicle developmental defects in zebrafish. Representative photomicrography showing altered Kupffer's vesicle inflation in both *cftr* -/- mutant and *cftr* morphant.

**Supplementary Figure 2. IL8-dependent neutrophil chemotaxis in *cftr*-defective zebrafish larvae**

(A) Tail transection was performed on control and *cftr* MO and mRNA levels of *cxcl8* gene determined by qRT-PCR in tail fin tissue at 2 and 4 hpi. Gene expression was normalized against *ef1a* and expressed as fold change over tail fin tissue from uninjured larvae (30 fins per replicate; mean relative ± SEM gene expression of 4 independent experiments; two-tailed Bonferroni t-test). (B) Mean number of recruited neutrophils into the otic cavity in response to mock or IL8 injection in control and *cftr* MO *TgBAC(mpx:EGFP)i114 larvae* monitored at 2 hpi (n=20; means ± SEM from 3 independent experiments; two-tailed Bonferroni t-test). Cell counts in control or CFTR-depleted animal show no significant difference (ns) in the average number of neutrophils recruited at IL8-injected site. (C) *TgBAC(mpx:EGFP)i114* controls, *cftr, Il8,* and double *cftr/il8* morphants were tail amputated and neutrophils at wounds were enumerated at 2 and 4 hpi (n=21; means ± SEM from 3 independent experiments; two-way ANOVA with Tukey post-test).

**Supplementary Figure 3. Neutrophil lifespan in *cftr*-defective zebrafish larvae**

3 dpf control and *cftr* MO *Tg(LysC:DSred)nz5* larvae were amputated and stained with acridine orange (OA) to label death cells. Representative confocal pictures of injured tails at 8 hpi (scale bars, 60 µm) revealing the proportion of dying neutrophils at the wound (white arrow). Dotted lines indicate the outline of the wounds.

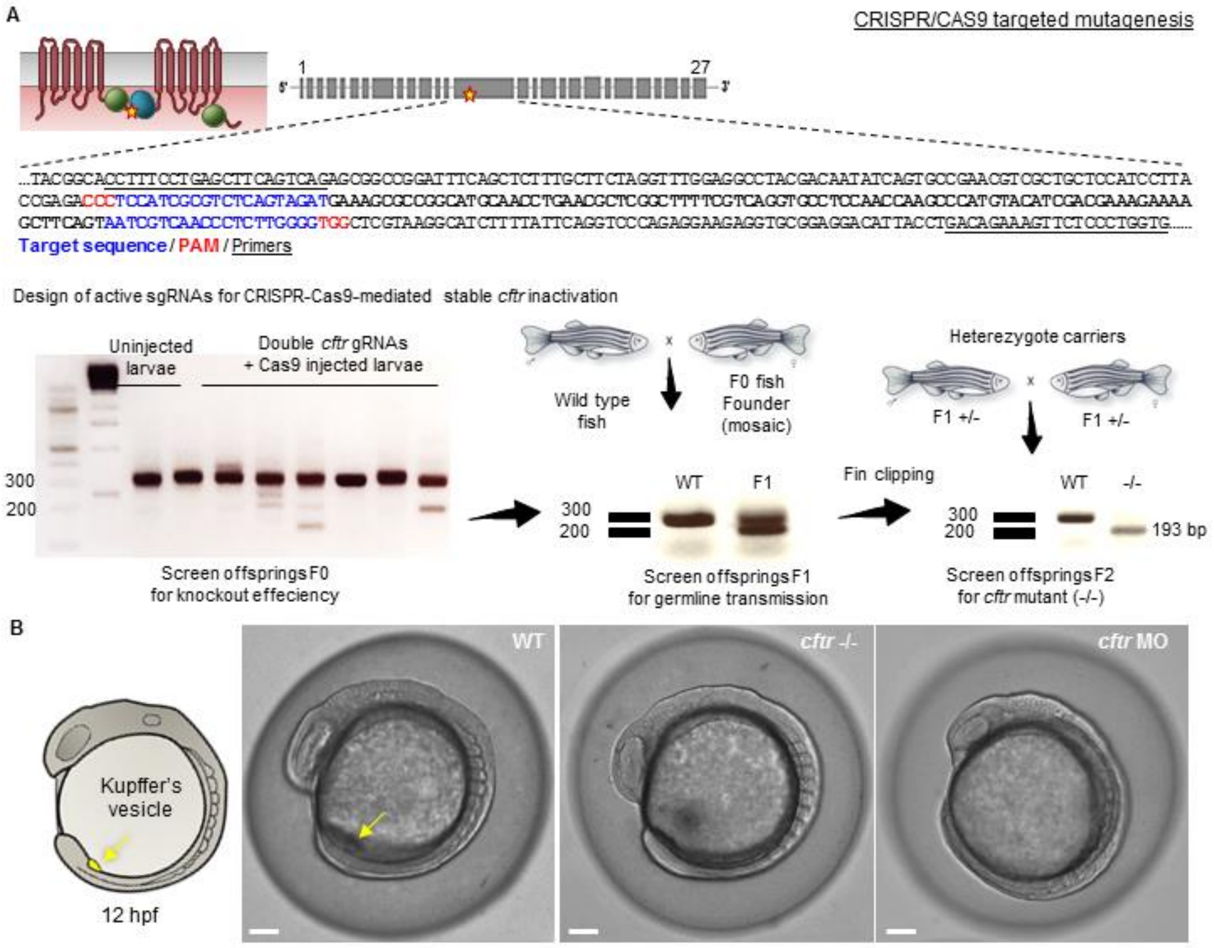
A
CRISPR/CAS9 targeted mutagenesis
1
27
5'
3'
...TACGGCACCTTTCCTGAGCTTCAGTCAGAGCGGCCGGATTTCAGCTCTTTGCTTCTAGGTTTGGAGGCCTACGACAATATCAGTGCCGAACGTCGCTGCTCCATCCTTA
CCGAGACCCTCCATCGCGTCTCAGTAGATGAAAGCGCCGGCATGCAACCTGAACGCTCGGCTTTTCGTCAGGTGCCTCCAACCAAGCCCATGTACATCGACGAAAGAAAA
GCTTCAGTAATCGTCAACCCTCTTGGGGTGGCTCGTAAGGCATCTTTTATTCAGGTCCCAGAGGAAGAGGTGCGGAGGACATTACCTGACAGAAAGTTCTCCCTGGTG.....
Target sequence / PAM / Primers
Design of active sgRNAs for CRISPR-Cas9-mediated stable cftr inactivation
Uninjected larvae
Double cftr gRNAs + Cas9 injected larvae
300
200
Screen offsprings F0 for knockout effeciency
Wild type fish
x
F0 fish Founder (mosaic)
WT
F1
300
200
Screen offsprings F1 for germline transmission
Fin clipping
Heterezygote carriers
F1 +/-
x
F1 +/-
WT
-/-
300
200
193 bp
Screen offsprings F2 for cftr mutant (-/-)
B
Kupffer's vesicle
12 hpf
WT
cftr -/-
cftr MO

S1

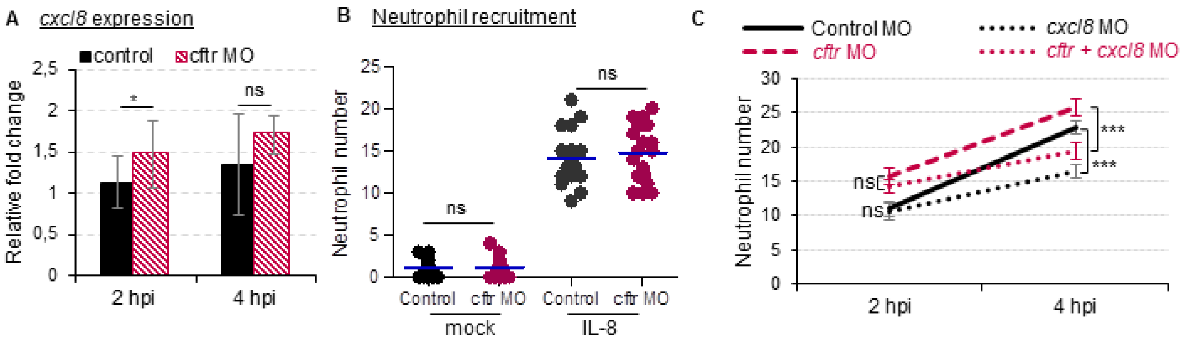
A cxcl8 expression
control
cftr MO
Relative fold change
2,5
2
1,5
1
0,5
0
*
ns
2 hpi
4 hpi
B Neutrophil recruitment
Neutrophil number
25
20
15
10
5
0
ns
ns
Control
cftr MO
Control
cftr MO
mock
IL-8
C
Control MO
cftr MO
cxcl8 MO
cftr + cxcl8 MO
Neutrophil number
30
25
20
15
10
5
0
ns
ns
***
***
2 hpi
4 hpi

S2

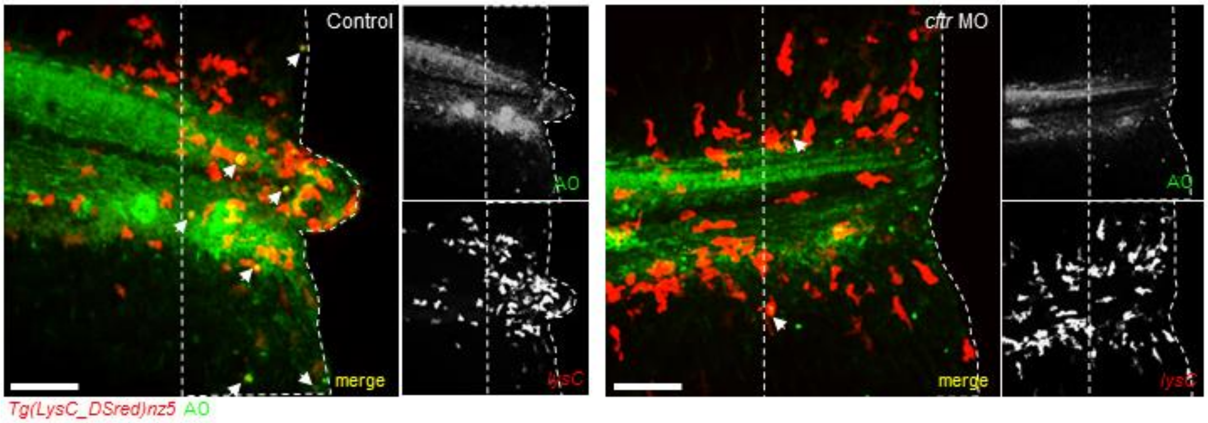
Control
AO
merge
lysC
cftr MO
AO
merge
lysC
Tg(LysC_DSred)nz5 AO

S3
